# Right atrial to left atrial area ratio on early echocardiography predicts long-term survival after acute pulmonary embolism

**DOI:** 10.1186/1476-7120-11-17

**Published:** 2013-05-31

**Authors:** Vincent Chow, Austin Chin Chwan Ng, Tommy Chung, Liza Thomas, Leonard Kritharides

**Affiliations:** 1 Concord Repatriation General Hospital and The University of Sydney, Sydney, Australia; 2 Liverpool Hospital, University of New South Wales, Sydney, Australia

## Abstract

**Background:**

Current guidelines recommend that transthoracic echocardiography (TTE) should be performed for acute risk stratification following acute pulmonary embolism (PE), but it is unclear whether the initial TTE can predict long-term outcome beyond six months. We sought to assess the potential of the initial right atrial (RA) to left atrial (LA) area ratio (RA/LA ratio) on TTE to predict long-term mortality in survivors of submassive PE.

**Methods:**

A derivation cohort comprised a previously reported group of 35 consecutive patients with acute PE who were intensively studied by serial TTE at 1, 2, 5 days, 2, 6, 12 and 26 weeks and RA/LA ratio related to long-term outcome. The Day 1 RA/LA ratio findings were then further related to long-term outcome in 158 patients followed for 3.6 ± 2.3 years.

**Results:**

In the derivation cohort, total mortality was 28.6% (n = 10) following a mean (±standard deviation) follow-up of 4.3 ± 1.9 years. The RA/LA ratio was highly dynamic, being increased at day 1, but normalised rapidly within 2–5 days of presentation and this was most marked amongst long-term non-survivors. A RA/LA ratio > 1.0 on day 1 was independently associated with a three-fold increase in long-term mortality on Kaplan-Meier analysis. Pooled analysis of 158 patient indicated that age, Charlson Comorbidity Index (CCI), simplified Pulmonary Embolism Severity Score (PESI), troponin T, day 1 RA/LA Ratio and pulmonary arterial systolic pressure (PASP) were univariate predictors of long-term mortality. Multivariate analysis identified Day 1 RA/LA Ratio (HR 1.7 per 10% increase,p = 0.002), CCI (HR 2.2 per 1 unit increase, p = 0.004) and age (HR 1.1, p = 0.03) as the only independent predictors of long-term mortality.

**Conclusion:**

A RA/LA Ratio >1.0 at presentation with acute PE was associated with a three-fold increased risk of long-term mortality. The RA/LA ratio on presentation with an acute PE is a simple, novel predictor of long-term survival.

## Introduction

Acute pulmonary embolism (PE) is common and associated with an early case fatality rate of 7-11% [1], and a reported 5-year cumulative mortality rate of up to 32% [2]. Clinical scores have been developed and validated in predicting short-term prognosis following acute PE [3-5]. Current guidelines recommend transthoracic echocardiography (TTE) should be performed for early risk stratification following acute PE [6]. After massive PE, right ventricular (RV) dysfunction on TTE, an enlarged right atrium (RA) with reduced left atrial (LA) size on computed tomography are prominent features and indicate worse acute prognosis [6-8].

Some predictors of adverse long-term outcomes after PE have been identified. These include initial troponin elevation, baseline comorbidities as assessed by the Charlson Comorbidity Index (CCI) and ongoing functional impairment post PE [9-14]. However, relatively little is still known about the long-term outcome of patients with PE. In particular, the ability of cardiac imaging parameters obtained early during acute PE to determine long-term prognosis in patients with submassive PE who survive to hospital discharge is particularly unclear [8]. In other conditions involving right heart strain such as pulmonary hypertension, RV dysfunction and RA dilation both confer significantly worse long-term prognosis [15,16]. While quantification of RV dysfunction is still evolving, RA size, either assessed as planimetry area or as RA/LA area ratio, is robust and easily measured on TTE or on CTPA and has been shown to correlate with the severity of the pulmonary arterial obstruction in submassive PE [17,18]. The greater the clot burden in the pulmonary arteries, the smaller the LA area and the larger the RA area become, thus resulting in an increased RA/LA area ratio. When patients present with an acute PE to the emergency department, the RA/LA area ratio, which can be easily measured, obtained instantaneously and is highly reproducible on echocardiography without the need for further post-processing as required by more complex strain assessment of right heart function, may be useful to the clinician. To the best of our knowledge, no study to date has examined the utility of the RA/LA area ratio, assessed early by TTE, in prognosticating the long-term mortality risk of patients post submassive PE.

The present study examined both the natural history changes to the RA/LA area ratio in patients with acute submassive PE, and additionally investigated the long-term prognostic significance of the RA/LA area ratio in these patients. Furthermore, we sought to determine whether a specific RA/LA area ratio cutoff on echocardiography is an independent prognostic marker for long-term outcomes in patients with submassive PE.

## Methods

### Derivation cohort

The design of the derivation cohort study has been described previously [19]. Thirty-five patients (mean age [±standard deviation] 63 ± 18years) with a clinical diagnosis of PE confirmed by a high probability ventilation/ perfusion (V/Q) pulmonary scintigraphy for PE were prospectively recruited from 2004 in a tertiary institution. Submassive PE was defined as involvement of 30% or greater lung parenchyma and massive PE was defined as involvement of 50% or greater lung parenchyma. Biochemical profiling (cardiac troponin-T, brain natriuretic peptide [BNP], D-dimer, C-reactive protein [CRP]) and serial TTE assessing RV function were performed on days 1, 2, 5 and weeks 2, 6, 12 and 26 following admission. All patients received systemic anticoagulation for a minimum of 6 months. The overall comorbid burden and short-term mortality risk of each patient was assessed and given a Charlson Comorbidity Index (CCI) score [20] and a simplified Pulmonary Embolus Severity Index (PESI) score respectively [3]. For all survivors, final follow-up visit for a full clinical and physical examination was completed in December 2010 (mean follow-up 4.3 ± 1.9years).

### Validation cohort

A larger validation cohort was derived from a previously published series of consecutive patients from 2001–2007 with outcome status and follow-up completed in July 2008 (mean follow-up 3.8 ± 2.6 years) [2]. From this group of 1023 patients, 123 were identified who underwent TTE on day 1 of admission and were further studied. These patients were used as the validation cohort. The age, gender, simplified PESI score, CCI score and clinical outcomes of patients who did not undergo TTE on day 1 in the validation cohort were not significantly different from those who did not undergo a TTE on day 1 (Additional file 1: Table S1). In both derivation and validation cohorts, the outcome status of all patients was first ascertained from the state (New South Wales, Australia) death registry. Surviving patients were contacted by telephone for outcomes with 100% complete follow-up. This study was approved by the institutional Human Research Ethics Committee.

### Echocardiography

A comprehensive TTE was performed with patients in the left lateral decubitus position (Vivid5, GE Medical Systems, Milwaukee, Wisconsin). Images were acquired from standard echocardiographic views in accordance with the American Society of Echocardiography recommendations [21]. All images were stored digitally and analyzed offline using commercially available software (GE EchoPac 3.1.3, Milwaukee, Wisconsin). TTE analyses were performed in batches on anonymized images with the operator (T.C) blinded to clinical details of each study. All measurements were averaged over 3 consecutive cardiac cycles.

In the derivation cohort, the RV was assessed by conventional 2-dimensional parameters that included: the ratio of RV and left ventricular (LV) end-diastolic diameters at the basal level (tricuspid annulus); the ratio of RV:LV end-diastolic areas; RV fractional area change [(RV end-diastolic area - RV endsystolic area)/RV end-diastolic area]; the ratio of right atrial (RA) and left atrial (LA) end-systolic areas. All areas were derived by planimetry from the apical 4-chamber view.

The pulmonary artery systolic pressure (PASP) was derived as the sum of the tricuspid regurgitant gradient obtained from continuous-wave Doppler and the right atrial pressure as estimated from the inferior vena cava [21]. The presence of McConnell’s sign (distinct akinesis of the mid RV free wall with preserved apical motion), a marker of RV strain and PE severity on echocardiography, was recorded [22]. RV longitudinal function was measured by determining the tricuspid annular plane systolic excursion (TAPSE). TAPSE was determined by M-mode measurement of the displacement of the lateral tricuspid annulus during systole and diastole in the apical 4-chamber view [23]. Intraobserver variability was assessed by randomly selecting 10 cases and repeating all analyses on three separate occasions with the operator blinded to previous results. The variability is presented as the coefficient of variation (CV).

In the validation cohort, the RA/LA area ratio, RV-RA pressure gradient, and the presence of RV dilatation and dysfunction were recorded [24]. In both cohorts, RV dilatation and/or dysfunction was defined as the presence of any of the following in accordance with published guidelines: ratio of RV and LV end-diastolic diameters >0.66; the ratio of RV:LV end-diastolic areas > 0.66; RV fractional area change < 0.35; TAPSE < 1.6 cm and/or presence of McConnell’s sign [24].

### Simplified PESI and CCI scores

The simplified PESI score is a validated index in the estimation of 30-day mortality in patients with acute PE. It incorporates the baseline demographics and comorbidities and the size of the pulmonary embolus based on hemodynamic status of the patient on admission. It is simpler to use and has similar prognostic accuracy and clinical utility as the original PESI score [3,25,26]. It comprised of six variables of equal weight (1 point per variable): age > 80 years old, history of cancer, history of chronic cardiopulmonary disease (chronic lung disease and/or heart failure), heart rate ≥110 beats per minute, systolic blood pressure <100 mmHg and arterial oxyhaemoglobin saturation level <90%. Patients with a simplified PESI score of <1 are classified as low risk and had a 30-day mortality of 1.0% compared to 10.9% mortality amongst high risk patients (simplified PESI score ≥1) [3].

The CCI is a summation score of the burden of comorbidities and it encompasses 17 different medical conditions including: myocardial infarction, congestive cardiac failure, peripheral vascular disease, cerebrovascular disease, dementia, chronic obstructive pulmonary disease, connective tissue disease, peptic ulcer disease, liver disease (mild vs. moderate to severe), diabetes (with or without organ damage), hemiplegia, moderate to severe renal disease, any tumor (within last 5 years), lymphoma, leukemia, metastatic solid tumor and acquired immunodeficiency syndrome [20]. It has been validated in predicting in-hospital mortality when applied to ICD-10 data [27]. A value of 0 indicates no comorbidity, while higher values represent an increasing burden of comorbid illnesses. A CCI score ≥3 is associated with high risk of mortality [28,29]. The simplified PESI and CCI scores were both tested for their prediction of long-term mortality post PE in our two cohorts.

### Statistical analysis

Data are summarized as frequencies and percentages for categorical variables. Continuous variables are presented as mean ± standard deviation. Comparison between groups used the unpaired *t* test for continuous variables and χ2 tests or the Fisher exact test for dichotomous variables. Kaplan-Meier survival methods were used to determine long-term mortality. A single multivariate Cox proportional hazards regression analysis was performed for the entire cohort to assess for univariate predictors of mortality. Only univariate variables with p < 0.05 were included in the multivariate Cox proportional hazards regression analysis. Linear mixed modelling was used to analyse the repeated data (RA/LA Area Ratio at different pre-specified times post PE) and if significant, t-test was performed at each pre-specified time to assess for significant difference in RA/LA Area ratio between long-term survivors and non-survivors. A p value of <0.05 was used as a cut-off for statistical significance. Statistical analysis was performed using GraphPad Prism 5.01 (GraphPad Software, San Diego, Calif), and SPSS (Version 16.0, SPSS Inc., Chicago, Ill).

## Results

Table 1 summarizes the baseline characteristics of the derivation cohort of 35 patients and the validation cohort of 123 patients. The mean extent of lung involvement assessed by pulmonary scintigraphy in the derivation cohort was 31 ± 23% consistent with submassive PE. This was supported by the absence of in-hospital deaths during the index PE admission, with 10 (28.6%) post discharge deaths over 4.3 ± 1.9 years of follow up with one death attributable to heart failure. In the validation cohort of 123 patients, there were no in-hospital deaths and 37 (30%) post discharge deaths over 3.4 ± 2.3 years of follow up with six deaths attributable to heart failure. In both cohorts, the mean simplified PESI score was <1.0, (0.9 ± 0.9 and 0.9 ± 1.0 for the derivation and validation cohorts respectively), thus implying these patients were at low risk of short-term mortality. In addition, the mean CCI score was <3.0 in both cohorts, suggesting a relatively low burden of comorbid disease. Thus both the derivation and validation cohorts had similar baseline and clinical characteristics as well as extent of lung involvement.

**Table 1:** Baseline characteristics

	**Derivation cohort (n = 35)**	**Validation cohort (n = 123)**	**p value**
Follow-up (years)	4.3 ± 1.9	3.4 ± 2.3	0.02*
Age, (years)	63 ± 18	68 ± 16	0.12
Male, no (%)	18 (51)	60 (49)	0.70
Comorbidities, no. (%)			
Atrial fibrillation	8 (23)	29 (24)	1.00
Diabetes	9 (26)	25 (20)	0.49
Heart Failure	2 (9)	21 (17)	0.11
Hypercholesterolemia	13 (37)	22 (18)	0.02*
Ischaemic heart disease	7 (20)	34 (28)	0.51
Malignancy	8 (23)	18 (15)	0.13
Stroke	4 (11)	3 (2)	0.04*
Charlson’s Comorbidity Index (CCI) Score	1.5 ± 1.8	1.6 ± 1.9	0.79
Clinical Outcomes			
Day 1 PASP, (mmHg)	33 ± 8	41 ± 16	0.006*
Simplified PESI score	0.9 ± 0.9	0.9 ± 1.0	0.88
Recurrent PE, no (%)	7 (20)	9 (7)	0.05
Death, no (%)	10 (29)	37 (30)	1.00

Values are presented as mean ± SD or absolute number.

*PE* Pulmonary embolism, *PESI* Pulmonary embolism severity index, *p < 0.05.

### Natural history of RA/LA area ratio after acute PE

To investigate whether the initial RA/LA area ratio, or its recovery, predicted long-term outcome, we first analysed the Day 1 RA/LA area ratio as well as its subsequent change for each patient over the 6 months follow-up period in the derivation cohort and related this to their long-term survival (Table 2). Long-term survivors had both a lower initial and more stable RA/LA area ratio (0.89 ± 0.26 and 0.83 ± 0.12 at day 1 and 6 months after PE). In contrast, non-survivors demonstrated acute RA dilation as evidenced by a RA/LA area ratio of 1.2 ± 0.37 at day 1, which decreased to 0.86 ± 0.11 by 6 months. However, no significant difference was present between the RA/LA area ratio of survivors and non-survivors when measured on day 2 after acute PE or thereafter. The apparent recovery in RA/LA area ratio on TTE between day 1 and 6 months was significantly greater in non-survivors than survivors (−0.42 ± 0.24 vs.- 0.01 ± 0.12, p < 0.001, respectively).

**Table 2:** Right to left atrial area ratio and its recovery over time

	**Overall (n = 35)**	**Non-survivors (n = 10)**	**Survivors (n = 25)**	**p value**
RA/LA Area Ratio D1	0.97 ± 0.32	1.20 ± 0.37	0.89 ± 0.26	0.01*
RA/LA Area Ratio D2	0.87 ± 0.22	0.96 ± 0.17	0.84 ± 0.23	0.23
RA/LA Area Ratio D5	0.89 ± 0.21	0.98 ± 0.19	0.84 ± 0.21	0.16
RA/LA Area Ratio Wk 2	0.83 ± 0.24	0.88 ± 0.31	0.80 ± 0.22	0.67
RA/LA Area Ratio Wk 6	0.82 ± 0.15	0.82 ± 0.21	0.82 ± 0.13	0.99
RA/LA Area Ratio Wk 12	0.90 ± 0.20	0.94 ± 0.22	0.89 ± 0.19	0.63
RA/LA Area Ratio Wk 26	0.84 ± 0.12	0.86 ± 0.11	0.83 ± 0.12	0.77

Values are presented as mean ± SD.

Linear mixed model showed significant difference in serial measurements of RA/LA area ratio over time between survivors and non-survivors, p = 0.041.

*D* Day, *Wk* Week, *LA* Left atrium, *RA* Right atrium, *p < 0.05.

**Figure 1:**
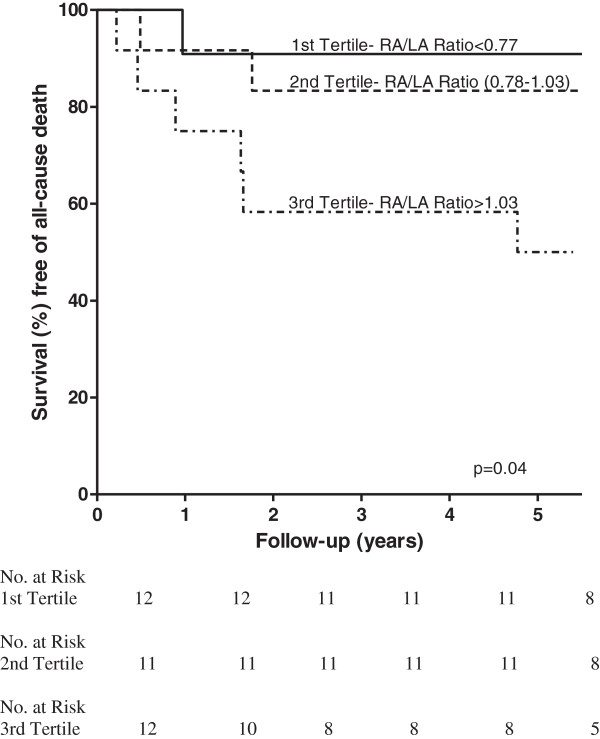
**Kaplan-Meier survival curve of the Derivation Cohort with RA/LA Area Ratio divided into tertiles.** Kaplan-Meier survival curve of the Derivation Cohort post-discharge following acute PE as a function of the RA/LA area ratio obtained on Day 1 post PE divided into tertiles. p = 0.04.

In order to identify a simple measure of RA/LA area ratio that may be of practical application, the RA/LA area ratios of the derivation cohort were analysed by tertiles. Kaplan-Meier survival analysis revealed a RA/LA area ratio >1.03 on day 1 admission TTE was associated with significantly worse long-term mortality (Figure 1). On this basis, an initial (day 1) RA/LA area ratio >1.0 was further investigated for its prognostic utility.

### Prognostic indicators in the combined cohort

**Figure 2:**
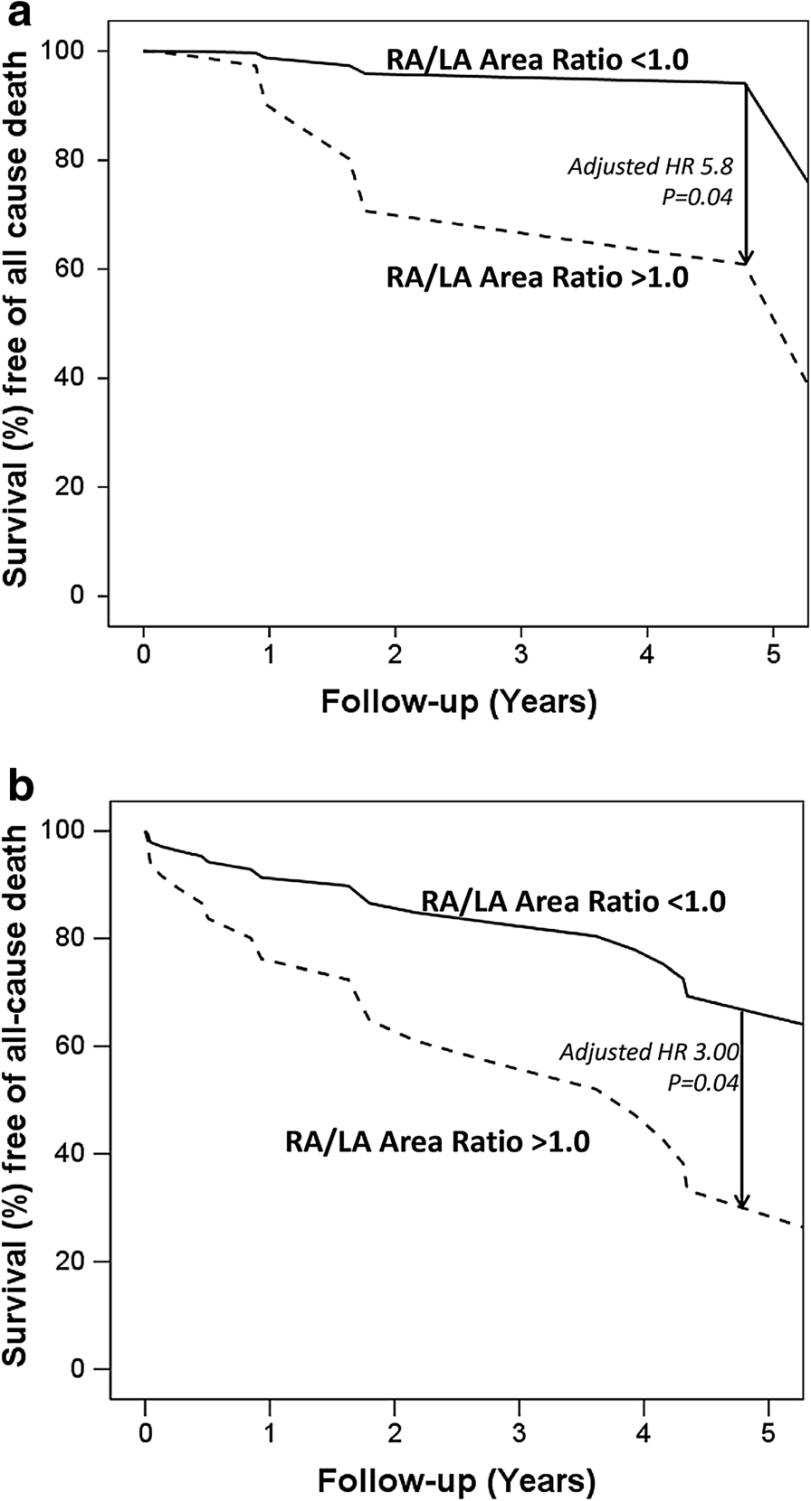
**Kaplan-Meier survival analysis for the Derivation Cohort. a**: Kaplan-Meier survival curve of the Derivation Cohort: post-discharge following acute PE utilising a cut-off of RA/LA area ratio of 1.0 on Day 1 post PE adjusted for other variables in the multivariate model. p = 0.04. **b**: Kaplan-Meier survival curve of the Validation Cohort: post-discharge following acute PE utilising a cut-off of RA/LA area ratio of 1.0 on Day 1 post PE adjusted for other variables in the multivariate model. p = 0.04.

In the derivation cohort, day 1 RA/LA area ratio >1.0 was an independent predictor of long-term mortality with a hazard ratio of 5.8, adjusted for CCI score, simplified PESI score and the presence RV dilatation and dysfunction (Figure 2a). In the validation cohort, the day 1 RA/LA area ratio was significantly increased in non-survivors than survivors (0.96 ± 0.22 vs. 0.84 ± 0.19, p = 0.009, respectively). A day 1 RA/LA area ratio >1.0 independently predicted long-term death with a hazard ratio of 3.0, adjusted for age, the CCI score, simplified PESI score, PASP and troponin T. (Figure 2b).

For the combined cohort, significant univariate predictors of death are shown in Table 3. These include measures of comorbidity (such as CCI score), measures of acute PE mortality risk (as measured by the simplified PESI score and Troponin T), age and measures of right heart dysfunction (including the day 1 RA/LA area ratio on day 1 and PASP).

**Table 3:** Univariate predictors of long-term mortality for the combined cohort (n = 158)

	**Hazard ratio**	**95% CI**	**p value**
**Baseline Characteristics**			
Age, per year increase	1.06	1.03-1.10	<0.001*
CCI score age unadjusted, per 1 unit increase	1.8	1.4-2.2	<0.001*
Simplified PESI score, per 1 unit increase	2.1	1.5-3.2	<0.001*
Troponin T, per 0.01 increase	1.1	1.03-1.25	0.008*
**RV parameters on Echocardiography**			
RA/LA Area Ratio (D1), per 10% increase	1.3	1.1-1.5	0.001*
RV dilation and dysfunction	0.6	0.3-1.2	0.16
PASP, per mmHg increase	1.04	1.01-1.07	0.007*

*CCI* Charlson Comorbidity Index, *D1* Day 1, *LA* Left atrium, *PASP* Pulmonary artery systolic pressure, *PESI* Pulmonary embolus severity index, *RA* Right atrium, *RV* Right ventricle. * p < 0.05.

Multivariate Cox-regression analysis using backward selection modelling identified the day 1 RA/LA area ratio (hazard ratio [HR] 1.7 per 10% increase, 95% confidence interval [CI] 1.2-2.4, p = 0.002), the CCI score (HR 2.2 per 1 unit increase, 95% CI 1.3-3.7, p = 0.004) and age (HR 1.1 per year increase, 95% CI 1.0-1.2, p = 0.03) as independent predictors of mortality (Table 4). Intraobserver variability was less than 10% for conventional echocardiographic parameters, which is consistent with reported literature [30,31]. In particular, the coefficient of variation (CV) for RA:LA end-systolic area was 5%.

**Table 4:** Multivariate backward selection model for combined cohort (n = 158)

	**Hazard ratio**	**95% CI**	**p value**
RA/LA Area Ratio (D1), per 10% increase	1.7	1.2-2.4	0.002*
CCI score age unadjusted, per 1 unit increase	2.2	1.3-3.7	0.004*
Age, per year increase	1.1	1.0-1.2	0.03*
Simplified PESI score, per 1 unit increase	2.0	0.9-4.4	0.08

*CCI* Charlson Comorbidity Index, *D1* Day 1, *LA* Left atrium, *PASP* Pulmonary artery systolic pressure, *PESI* Pulmonary embolus severity index, *RA* Right atrium, *RV* Right ventricle. * p < 0.05.

## Discussion

The ability of echocardiography to predict long-term mortality after a submassive PE is not clear. Echocardiography is recommended during the acute management of PE to assess RV dysfunction so as to further stratify non-hemodynamically compromised patients into intermediate or low-risk prognostic categories [6]. In the current study, we report for the first time that the day 1 RA/LA area ratio on echocardiography in patients with acute submassive PE predicted long-term mortality in both the derivation and validation cohorts. A day 1 RA/LA area ratio >1.0 on presentation conferred at least a three-fold increase in long-term mortality. This was independent of the expected predictive value of the Charlson Comorbidity Index and simplified PESI Scores.

Pulmonary embolism causes an increase in RV afterload. The RA and RV are thin walled and low pressure chambers, unlike the LV, and have limited capacity to compensate to the elevated RV wall tension. This results in RV dilation and dysfunction and worsening tricuspid regurgitation.

Previous studies have shown the presence of RV dysfunction post PE, in particular an increased RV/LV ratio, by both computed tomography pulmonary angiography (CTPA) and TTE, correlated with clinical outcomes and short-term mortality [32-37]. However in other studies, no clear association could be demonstrated [38,39]. This discrepancy may be due to absence of an uniformly accepted standard for measuring the ventricular volumes on CT and the lack of electrocardiographic gating of the CTPA [40].

The RA/LA area ratio reflects the integration of multiple haemodynamic parameters including diastolic and systolic function of the RV, and filling volumes and pressures of the LA and LV. Amongst the general population without a history of pulmonary embolism, the RA/LA area ratio has been reported to be 0.81 ± 0.15 [41]. A raised RA/LA area ratio has previously been shown by our group to correlate with the extent of pulmonary artery occlusion after PE [18]. In acute PE, the sudden increase in RV afterload can lead to RV and RA dilation. This additionally impairs left- atrial and ventricular diastolic filling due to deviation of the inter-atrial and inter-ventricular septae leftwards. These changes have been observed on CT scans in patients with massive PE [42,43]. A relative increase in RA and RV volumes coupled with decrease in LA and LV volumes leads to an increased RA/LA area ratio. Our results indicate that these changes are also evident in submassive PE without hemodynamic instability and are dynamic.

While it may be expected that RA/LA area ratio may relate to early outcome, the association with long-term outcome as demonstrated in the present study is novel. A particularly important finding of our study is that there is an acute recovery of the RA/LA area ratio and that this recovery does not predict a favourable long-term outcome. Therefore, TTE performed on or after day 2 of PE presentation in earlier studies are likely to underestimate the prognostic utility of the RA/LA area ratio. Pathophysiologically, our study indicates that it is the initial cardiac response to the thromboembolic insult and not the subsequent recovery that predicts long-term outcome. That the RA/LA area ratio is highly dynamic and responds acutely to volume and pressure change has been shown previously after percutaenous atrial septal defect closure. Within 24 hours of closure, Kelly et al. observed significant and immediate decrease in the RA/LA area ratio compared with baseline TTE, despite an extended period prior with significant right sided volume overload [41]. This is due to the immediate volume and pressure unloading of the right heart, with complete normalization of the RA/LA area ratio at early follow-up. We postulate a similar mechanism occurs in patients with acute PE where right sided volume changes occur more acutely and therefore making these changes more reversible. Following the commencement of anticoagulation therapy and the resulting reduction in thromboembolic burden in the pulmonary vasculature, there is an immediate volume and pressure unloading of the right heart with improvement in the RA/LA area ratio within 24 hours. Presumably, these patients with elevated RA/LA area ratio on Day 1 indicate a reduced cardiac-pulmonary capacity to cope with increased RV afterload, even if there is normalisation of the RA/LA area ratio over the first week of treatment independent of the initial PE size as calculated by the simplified PESI score.

The RA/LA area ratio is simple to measure and a cutoff of day 1 RA/LA area ratio >1.0 may be a practical guide for physicians to identify patients who are at increased risk of death in the long-term following submassive PE. In particular, we have identified 17 of the 37 (46%) deaths in the validation cohort were cardiovascular or recurrent pulmonary embolus deaths which may have been preventable. These patients may benefit from long term follow-up and closer surveillance [6,7] to ensure appropriate anticoagulation therapy is provided and that there is normalisation of the RA/LA area ratio and RV function with normalisation of pulmonary haemodynamics.

The CCI is a summation score of the burden of Comorbidities [20] shown to be very useful in prognosticating the outcome of patients suffering from diseases including heart failure [44], endocarditis [45] and cancer [46]. In our current study, we confirmed the CCI is an independent predictor of long-term death following submassive PE. Among the included comorbidities in the CCI are cardiovascular diseases, chronic pulmonary diseases and malignancies. These comorbid illnesses have previously been found to be present in patients presenting with venous thromboembolism [47] and were predictors of long-term functional impairment[48] and survival [14]. We have shown for the first time that a simple and easily obtained echocardiographic parameter, the RA/LA area ratio, is an independent predictor of long-term survival following acute PE.

### Limitations

This study is limited by its sample size. This may explain why cardiac troponin-T and BNP levels, which were raised amongst the non-survivors, were not independent predictors of long-term mortality following multivariate analysis in our combined cohort. We have recently demonstrated a concentration-dependent relationship between cardiac troponin-T elevation following acute PE and long-term mortality [13] and it will be important to extend the present study into larger patient cohorts to directly compare RA/LA area ratio and biochemical parameters in predicting long-term outcome.

## Conclusion

In summary, patients suffering from pulmonary embolus have significantly increased long-term mortality. The early echocardiographic RA/LA area ratio is a simple, robust and easily reproducible measurement, which independently predicted long-term mortality amongst PE patients. A day 1 RA/LA area ratio >1.0 is independently associated with a three-fold increase in long-term mortality and this novel echocardiographic parameter may aid the long-term risk stratification following acute submassive PE.

## Competing interests

The authors declare that they have no competing interests.

## Authors’ contribution

VC, AN, TC, LZ, LK planned the study. VC and TC performed measurements and statistical analysis. All took major part in the writing and reviewing of the article. All authors have read and approved the final manuscript.

## Supplementary Material

Additional file 1: Table S1. Comparison of baseline characteristics within the validation database between patients who did or did not undergo Day 1 TTE. Click here for file
