# Supplementary material for: Right atrial to left atrial area ratio on early echocardiography predicts long-term survival after acute pulmonary embolism
Source: Cardiovasc Ultrasound. 2013 May 31;11:17. doi: 10.1186/1476-7120-11-17 (PMC3673888; doi:10.1186/1476-7120-11-17)
Supplement: Additional file 1: Table S1. — Comparison of baseline characteristics within the validation database between patients who did or did not undergo Day 1 TTE. [file 1476-7120-11-17-S1.doc]

**Supplementary Table 1:
Comparison of baseline characteristics within the validation database between patients who did or did not undergo Day 1 TTE**

|  | **Day 1 TTE performed**  **(n=123)** | **Day 1 TTE not performed**  **(n=900)** | **p value** |
| --- | --- | --- | --- |
| Follow-up (years) Age, mean ± SD | 3.4 ± 2.3 68 ± 16 | 3.9 ± 2.6 68 ± 16 | 0.04*  0.96 |
| Male, no (%) | 60 (49) | 397 (44) | 0.34 |
|  |  |  |  |
| Comorbidities, no.(%) |  |  |  |
| Atrial fibrillation | 29 (24) | 128 (14) | 0.01* |
| Diabetes | 25 (20) | 133 (15) | 0.11 |
| Heart Failure | 21 (17) | 112 (12) | 0.15 |
| Hypercholesterolemia | 22 (18) | 120 (14) | 0.17 |
| Ischaemic heart disease | 34 (28) | 176 (20) | 0.05 |
| Stroke | 3 (2) | 30 (3) | 0.79 |
| Charlson’s Comorbidity Index (CCI) Score | 1.5 ± 1.8 | 1.6 ± 1.9 | 0.79 |
|  |  |  |  |
| Clinical Outcomes |  |  |  |
| Simplified PESI score | 0.9 ± 1.0 | 0.9 ± 0.9 | 0.76 |
| Recurrent PE | 9 (7) | 85 (9) | 0.51 |
| Death | 37 (30) | 326 (36) | 0.19 |

Values are presented as mean ± SD or absolute number.
PE = Pulmonary embolism, PESI= Pulmonary embolism severity index, TTE= Transthoracic echocardiography, *p<0.05.
